# BKM-120 (Buparlisib): A Phosphatidyl-Inositol-3 Kinase Inhibitor with Anti-Invasive Properties in Glioblastoma

**DOI:** 10.1038/srep20189

**Published:** 2016-02-05

**Authors:** Maria-Carmela Speranza, Michal O. Nowicki, Prajna Behera, Choi-Fong Cho, E. Antonio Chiocca, Sean E. Lawler

**Affiliations:** 1 Harvey Cushing Neurooncology Laboratories, Department of Neurosurgery, Brigham and Women’s Hospital, Harvard Medical School. Boston MA 02115, USA

## Abstract

Glioblastoma is an aggressive, invasive tumor of the central nervous system (CNS). There is a widely acknowledged need for anti-invasive therapeutics to limit glioblastoma invasion. BKM-120 is a CNS-penetrant pan-class I phosphatidyl-inositol-3 kinase (PI3K) inhibitor in clinical trials for solid tumors, including glioblastoma. We observed that BKM-120 has potent anti-invasive effects in glioblastoma cell lines and patient-derived glioma cells *in vitro*. These anti-migratory effects were clearly distinguishable from cytostatic and cytotoxic effects at higher drug concentrations and longer durations of drug exposure. The effects were reversible and accompanied by changes in cell morphology and pronounced reduction in both cell/cell and cell/substrate adhesion. *In vivo* studies showed that a short period of treatment with BKM-120 slowed tumor spread in an intracranial xenografts. GDC-0941, a similar potent and selective PI3K inhibitor, only caused a moderate reduction in glioblastoma cell migration. The effects of BKM-120 and GDC-0941 were indistinguishable by *in vitro* kinase selectivity screening and phospho-protein arrays. BKM-120 reduced the numbers of focal adhesions and the velocity of microtubule treadmilling compared with GDC-0941, suggesting that mechanisms in addition to PI3K inhibition contribute to the anti-invasive effects of BKM-120. Our data suggest the CNS-penetrant PI3K inhibitor BKM-120 may have anti-invasive properties in glioblastoma.

Glioblastoma (GBM) is the most common primary malignant glial brain tumor with an annual incidence of 3.5/100,000 in the US^1^. GBM is also one of the most lethal human cancers with 12–15 months median survival and 5-year survival of just 5%^2^. The current standard-of-care was established a decade ago and consists of maximal safe surgical resection followed by concomitant radio- and chemotherapy^3^.

Infiltration of normal brain parenchyma is a defining characteristic hallmark of GBM. These cells render GBM surgically incurable and 90% of patients develop new lesions within 2–3 cm of the primary tumor or at distant sites within the brain^1^. Moreover, invasion may be increased further by commonly used therapies including radiation and avastin^4^. At present, there are no anti-invasive drugs available clinically, and this is a widely acknowledged clinical challenge. The identification of a CNS-penetrant anti-invasive drug may have an important clinical impact in GBM.

The phosphatidyl-inositol-3 kinase (PI3K) pathway is frequently deregulated in cancer^1^,^5^. PI3K is a lipid kinase that transduces growth-promoting extracellular signals and controls downstream effectors implicated in malignancy^6^,^7^. PI3K is activated in the majority of GBMs due to constitutive receptor tyrosine kinase activation as well as inactivating mutations/deletions of PTEN (33%) or activating PI3K mutations (17%)^1^,^8^,^9^. Small molecule PI3K inhibitors are under investigation in oncology clinical trials^6^.

BKM-120 (Buparlisib), a dimorpholino pyrimidine derivative, is an oral pan-class I PI3K inhibitor that penetrates the blood-brain barrier (BBB)^6^. It is in clinical trials for solid tumors including GBM, and has anti-proliferative and pro-apoptotic effects in GBM cell lines independent of PTEN or EGFR status^10^. BKM-120 selectively inhibits PI3K isoform α (PIK3CA) with an *in vitro* IC_50_ of 35 nM, and inhibits other PI3K paralogs with an IC_50_ range of 108–348 nM^6^. BKM-120 induces a G2-M cell cycle arrest *in vitro*, blocks VEGF-induced neo-vascularization *in vivo* and its activity persists in the presence of activating PIK3CA mutations^11^. Off-target effects have been reported through direct binding of BKM-120 to tubulin causing microtubule polymerization^12^.

PI3K plays a role in cell migration in some cell types^5^,^13^. Therefore, because of the need for clinically applicable anti-invasive approaches in GBM, and the fact that BKM-120 penetrates the BBB, we investigated its effects on GBM cell migration. We found that BKM-120 caused a dose-dependent, reversible blockade of GBM invasion and migration *in vitro* in GBM cell lines and glioma stem-like cells (GSCs). *In vivo* studies showed a marked reduction in invasive tumor spread in mice bearing orthotopic xenografts treated with BKM-120. Mechanistically, BKM-120 treatment led to a reduction in focal adhesions and microtubule treadmilling, which may contribute to its anti-migratory effects. These data suggest that BKM-120 is a candidate anti-invasive drug for GBM therapy.

## Materials and Methods

### Cell culture and reagents

Glioma stem cell (GSC) lines G9, G33, G35, G146 and G157 were described previously^14^,^15^, and BT145 was obtained from the BWH Neuropathology core. All samples were collected according to IRB approved protocols, and maintained as described^16^. U87 and U251 GBM cell lines and astrocytes were provided by ATCC (Manassas, VA), U1242 cells were a gift from Dr. James Van Brocklyn (The Ohio State University) and grown in DMEM (Life Technologies) with 10% fetal bovine serum (FBS, Sigma-Aldrich) and 1% penicillin-streptomycin. The isolation of the human fetal NSCs SCP27 (a kind gift from Dr Brian Kaspar, Nationwide Children’s Hospital, Columbus, OH) were reported previously^17^. Copepod GFP (copGFP) marker protein was transduced using pCDH (System Biosciences, Mountain View, CA). Cells transfected with Paxillin-GFP (Addgene, 15223) and EB1-GFP (Addgene, 17234; provided by Dr Hiroshi Nakashima, Brigham and Women’s Hospital) plasmids were plated after 48 hours in Nunc™ Lab-Tek™ II Chambered Coverglass 8-well plates (Thermo Fisher Scientific, Inc.) and treated with vehicle and drugs. BKM-120 and GDC-0941 were purchased from Selleckchem (Houston, TX).

### Cell invasion and migration assays

For 3D spheroid cultures, 5,000 cells/well were cultured in Corning Ultra-Low Attachment Surface 96 well plates (Corning Inc., Corning, NY) in 100 μl medium. After 24 hours, the medium was replaced with 50 μl of collagen I (Advanced BioMatrix, Inc San Diego, CA, USA) and neutralized to pH 7.5 using 1N NaOH and supplemented with FCS, penicillin-streptomycin, and 5 x DMEM. After polymerization, the collagen was overlaid with 50 μl of medium containing drug or vehicle. Wound-healing assays were performed as described^16^. Transwell assays were performed using 3 μm pore size fluoroblok inserts (Corning Inc., Corning, NY). Poly-ɛ-caprolactone nanofibers (Nanofiber Solutions, Columbus, OH) were prepared and quantitated as described^18^.

### Cell viability, cell-substrate adhesion and cell-cell adhesion assays

Cell viability and proliferation were measured using PrestoBlue reagent according to the manufacturer’s instructions (Life Technologies, Grand Island, NY). Cell-substrate adhesion assays were performed with adherent G9-copGFP cells in paired 96 well plates. Cells were treated with vehicle (DMSO) as control or BKM-120 using four replicates at each time point for each condition. Drug and vehicle were washed out every 30 min and replaced with fresh medium. One plate was used for microscopic observation; cells were fixed with 4% PFA, treated with 5% normal goat serum and 0.5% Triton-x-100 in PBS for 1 hour at room temperature, and stained with DAPI. The second plate was used to evaluate cell-substrate adhesion with the CellTiter-Glo^®^ Luminescent Cell Viability Assay (Promega, Madison, Wisconsin, USA) according to the manufacturer’s instructions. Cell-cell adhesion was evaluated using a G9-copGFP single cell suspension treated with DMSO or BKM-120 and placed in a circular shaker in the cell incubator. At prefixed time-points 50 μl of each suspension was transferred to a 96-well plate and fixed with 4% PFA. Each experiment was performed in 4 replicates.

### *In vivo* studies, drug and tissue preparation

2 × 10^5^ G9-copGFP or U1242 cells were stereotactically injected into nude mouse brains (2 mm right lateral, 1 mm frontal to the bregma and 3 mm deep) as described^15^. A short timeline was chosen in order to show a clearer anti-invasive effect, therefore treatments started on day 4 and on the first day of injection three mice were sacrificed to show the tumors at time 0. Starting on day 7 for the G9-copGFP and day 4 for the U1242 post-implantation five mice per group were treated for four days by daily gavage with vehicle (NMP/PEG300, Sigma-Aldrich), 2 mg/kg or 20 mg/kg of BKM-120. Brains were harvested on day 10 (G9-copGFP) and on day 7 (U1242), placed in 4% PFA for 24 hours, followed by 30% sucrose for 48 hours, and frozen. Sections (30 μm) were blocked with 5% donkey serum/0.5% TX100/ PBS for 1 hour at room temperature, followed by Vimentin SP20 (1:200 O/N, ab16700 Abcam, Cambridge, MA, USA) and Hoechst 33342 staining (1:3000, H3570 Life Technology, Grand Island, NY, USA). BKM-120 was formulated in NMP/PEG300 (10/90). Solutions were freshly prepared on each day of dosing by dissolving the drug first in NMP and then by adding the remaining volume of PEG300. The human equivalent dose (HED) of BKM-120 was calculated according to the US Food and Drug Administration method previously described by Shannon *et al*.^19^,^20^.

### Microscopy

Nikon Eclipse Ti, Nikon TE2000 and Zeiss LSM710 confocal microscope systems with on-stage incubators for live cell imaging were used. Data were edited/quantified using ImageJ (http://rsb.info.nih.gov/ij/), Microsoft Excel and Prism 6 (GraphPad Software Inc., San Diego, CA).

### Statistical analysis

Two sample t tests (adjusted with Bonferroni’s method), one-way and two-way ANOVA (Greenhouse-Geisser correction) were used. IC_50_ value was calculated as the drug concentration required to reach an inhibitory effect of 50% compared to the vehicle control.

## Results

### BKM-120 inhibits GBM spheroid invasion in a dose-dependent manner

**Figure 1:**
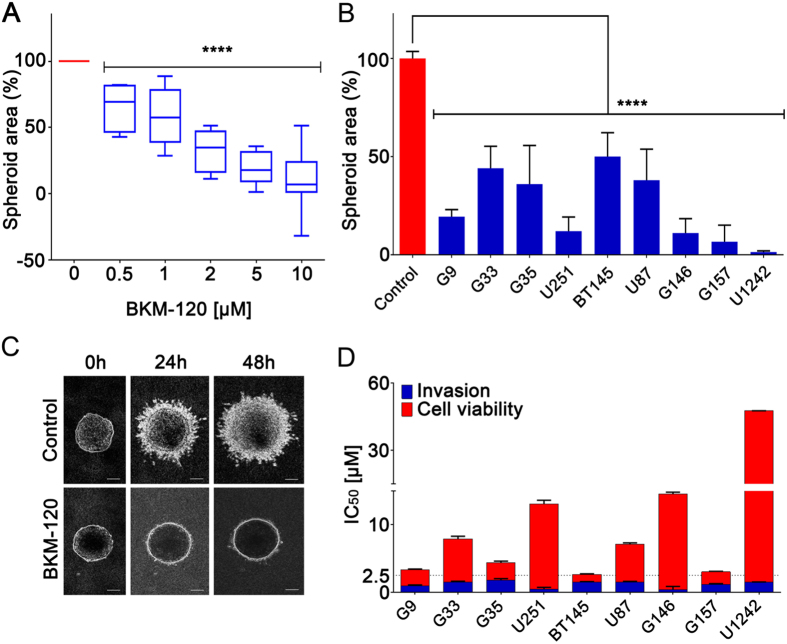
BKM-120 inhibits invasion in a dose-dependent manner in GBM cell lines. (**A**) The graph represents an average of the effects of BKM-120 on nine different GBM cell lines and GSCs. **(B**) Effect of increasing concentrations of BKM-120 in spheroid invasion assays in a panel of GBM cell lines. Invasion was measured after 48 hours of BKM-120 treatment and is expressed as sphere area as a percentage of controls. (**C**) Image of G9 spheroids after treatment with 2 μM BKM-120 (bar = 100 μm) (lower panel) compared with controls (upper panel). (**D**) Invasion (blue) and cell viability (red) IC_50_ values for BKM-120. IC_50_ was calculated using data from a range of BKM-120 concentrations. Measurements were obtained at 48h of treatment. One-way ANOVA was used for statistical analysis (****p < 0.0001).

PI3K has been previously linked to cell migration in several studies^5^,^13^, and is a major oncogenic signaling molecule in GBM^1^,^5^. Initially, we examined the effects of the CNS-penetrant selective class I PI3K inhibitor BKM-120 in 3D spheroid invasion assays on a panel of GBM cell lines, including low passage GSCs, neural stem cells and astrocytes (Fig. 1A, Supplementary Fig. 1A). BKM-120 inhibited GBM spheroid invasion in a dose-dependent manner, with a significant blockade observed at 1–2 μM (Fig. 1A). Invasion was inhibited by at least 50% in all cell lines at 2 μM (Fig. 1B), and in highly sensitive cell lines (G9, U251, G157, G146 and U1242) invasion was strongly blocked at this concentration (>90% inhibition). Figure 1C shows a clear blockade of invasion in G9-copGFP cells treated with 2 μM BKM-120 over 48 hours. Cell proliferation assays showed that across the cell line panel the IC_50_ values for cell viability were higher than those for invasion, confirming the ability of BKM-120 to specifically inhibit GBM invasion (Fig. 1D, Supplementary Fig. 1A).

### BKM-120 inhibits GBM cell migration in multiple *in vitro* assays and its effects are reversible

**Figure 2:**
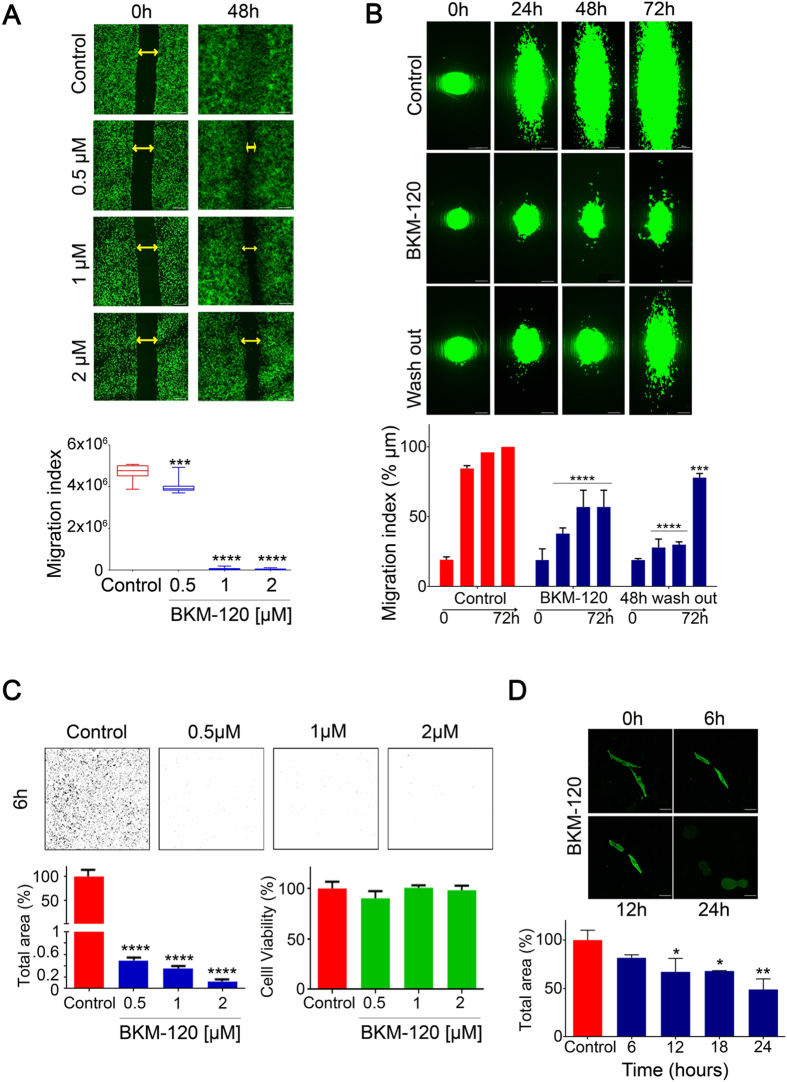
Impact of BKM-120 on GBM cell migration and morphology. **(A**) Time-lapse images of U251-GFP GBM cells in wound-healing assays performed on U251-copGFP with increasing concentrations of BKM-120 (0.5, 1 and 2 μM) over 48 h. Controls were treated with DMSO. The gap is indicated by double arrows and the migration index was measured as total area of the gap (bar = 100 μm). (**B**) Time course over 72 hours on nanofiber scaffolds of G9-copGFP GBM cells treated with DMSO or BKM-120 (2 μM). The wash out performed at 48 hours demonstrates recovery of cell migration after drug removal. The lower graph indicates percentage of migration index compared to controls at 72 hours (100%) (bar = 100 μm). (**C**) Dose-dependent effect of BKM-120 on U251 GBM cells in transwell assays after 6 hours. The images show the ImageJ generated masks used to quantify the cell migration. Cell viability assays were performed on the same cells at the same time point using PrestoBlue. (**D**) Cell morphology. Time course of U251-copGFP GBM cell shape changes after treatment with 2 μM BKM-120 (bar = 50 μm). The graph indicates the percentage of total area compared to time 0h at 6, 12 and 24 hours (100%) (bar = 100 μm). One-way ANOVA was used for statistical analysis (*p < 0.05, **p < 0.01, ***p < 0.001, ****p < 0.0001).

We then investigated the effects of BKM-120 on GBM cell migration in a range of additional *in vitro* migration assays. Wound-healing “scratch” assays confirmed that BKM-120 significantly inhibited GBM cell migration in a dose-dependent manner in U251 and G9 cells (Fig. 2A, Supplementary Fig. 2A) at similar concentrations to those observed in spheroid assays. We then examined the effect of BKM-120 on GBM cell migration on aligned nanofiber scaffolds, which we previously developed as a topographic substrate for cell migration^18^. Cell migration was inhibited in a dose-dependent manner in multiple cell lines similar to above (Supplementary Fig. 2B), and was strongly inhibited at 2 μM BKM-120 (Fig. 2B). Importantly, using the nanofiber assay we confirmed that the effects of BKM-120 were not due to irreversible cytotoxicity; a drug wash-out experiment performed after 48h treatment with 1 μM BKM-120 (Supplementary Fig. 2C,D) and 2 μM BKM-120 showed that the anti-migratory effects of BKM-120 were reversible (Fig. 2B). Transwell assays performed on U251-copGFP (Fig. 2C) and G9-copGFP cells at the initial time-point (6 hours) and at later time-points (24 and 48 hours, data not shown) showed a similar block of migration. In summary, these data demonstrate that BKM-120 potently and reversibly inhibits migration in a dose-dependent manner. Moreover, cell morphology changes induced by BKM-120 observed during the nanofiber experiment were confirmed in U251-copGFP cells treated with 1 μM BKM-120, in which cells became completely rounded up after 24 hours (Fig. 2D).

### BKM-120 blocks GBM cell-adhesion

**Figure 3:**
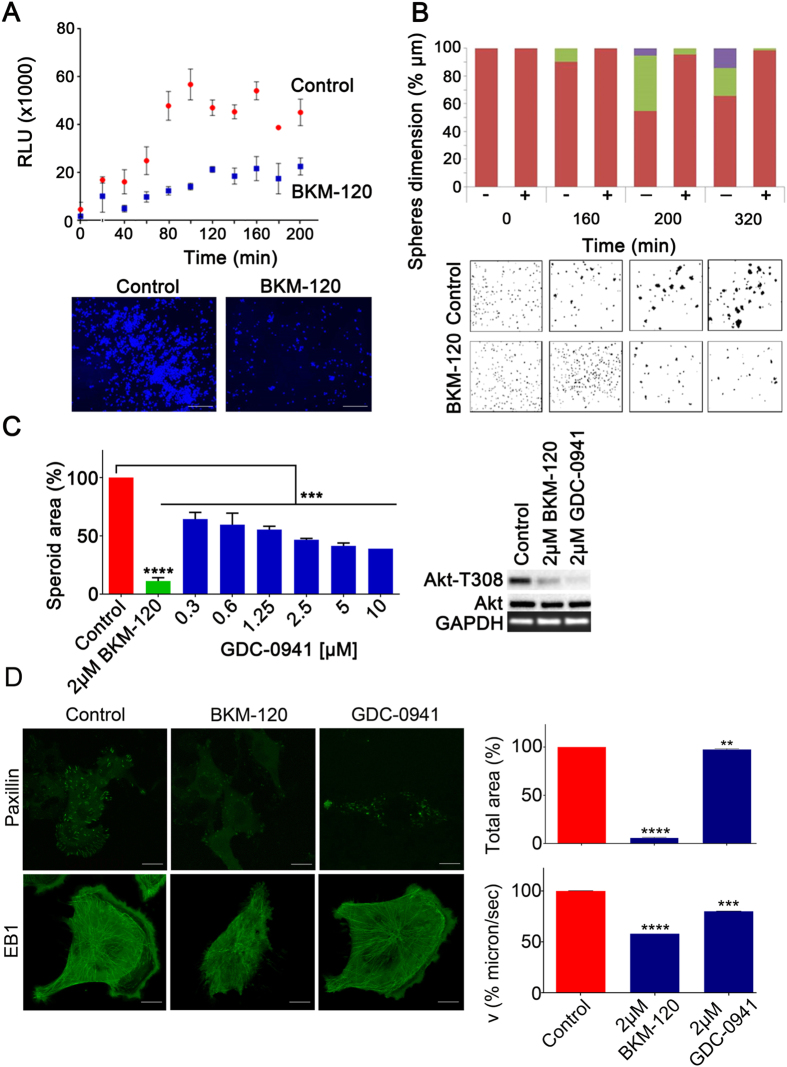
BKM-120 reduces GBM cell adhesion, focal adhesions and microtubule dynamics. **(A**) Cell-substrate adhesion was evaluated using G9-copGFP GSCs as described in Materials and Methods. Measurements were taken as luminescence output, RLU (relative light units), every 20 minutes over a time course of 200 minutes. The lower panel shows DAPI staining of G9-copGFP GSCs at the final time-point (200 min, bar = 500 μm). (**B**) Cell-cell adhesion in G9-copGFP GSCs. Sphere dimensions are indicated as a percentage relative to controls at the indicated time-points. The sphere dimension range is color coded in red for <3000 μm (1), green for 3000 - 6000 μm (2) and violet > 6000 μm (3). (**C**) Left panel: **s**pheroid invasion assay on G9-copGFP GSCs with 2 μM of BKM-120 and a range of GDC-0941 concentrations. Invasion was measured after 48 hours and is expressed as percentage of spheroid area compared to controls. Right panel: Akt phosphorylation levels were measured by Western blot after 30 minutes treatment with 2 μM of BKM-120 or GDC-0941. (**D**) U251-paxillin-GFP images (bar = 20 μm) and U251-EB1-GFP time-lapse (bar = 10 μm) at 24h after DMSO, BKM-120 or GDC-0941 treatment. The total area of the paxillin-GFP signal and the velocity (microns/sec) of EB1 were measured using ImageJ. Each measurement was made in triplicate. One-way ANOVA was used for statistical analysis (**p < 0.01, ***p < 0.001, ****p < 0.0001).

We then investigated the impact of BKM-120 on GBM cell-substrate and cell-cell adhesion *in vitro*. Cell-substrate adhesion in G9-copGFP cells was significantly inhibited by 2 μM BKM-120 and confirmed by microscopic imaging after 200 min (p = 0.009) (Fig. 3A). Cell-cell adhesion was measured using an aggregation assay in suspension culture, by setting three size ranges and counting the number of cell clusters in each specific range dimension. This assay therefore allows discrimination between aggregates of different size. At time 0 all the cell clusters, both in control or BKM-120 treated samples, have a size <3000 μm, indicating small cell-cell aggregates and single cells. After 160 min, 10% of control cells had formed aggregates with size in the 3000–6000 μm range, whereas all of the BKM-120 treated cells remained in the smaller range (p < 0.001). This pattern continued over the full time course of the experiment (Fig. 3B). Taken together these data demonstrate that BKM-120 blocks cell-substrate and cell-cell adhesion suggesting it affects the ability of cells to form robust contacts.

### PI3K-independent effects of BKM-120 contribute to the migratory phenotype

We then investigated the anti-invasive effects of another potent selective PI3K inhibitor, GDC-0941^21^, that is in phase I clinical studies for advanced solid tumors^22^. This showed a modest effect on cell invasion compared with BKM-120 even though PI3K inhibition was stronger after GDC-0941 treatment as shown by Akt phosphorylation (Fig. 3C). GDC-0941 did not fully block migration even at concentrations as high as 10 μM (Fig. 3C), cell-substrate adhesion in the presence of 2 μM GDC-0941 was not significantly altered compared with controls (data not shown, p = 0.1) and was significantly less compared with BKM-120 (p = 0.009). *In vitro* kinase selectivity profiling revealed no significant differences between BKM-120 and GDC-0941 in a panel of 140 protein kinases and 15 lipid kinases (Supplementary Table 1). Similarly, we did not see any differences between the effects of either drug in an ELISA-based array of 39 major phospho-proteins (Supplementary Figure 3A). In fact, our Western blotting data suggests that GDC-0941 has a stronger inhibitory effect on PI3K than BKM-120. Overall, these data suggest that PI3K inhibition alone is not sufficient to block glioblastoma cell migration.

To better understand these observations and the potential PI3K-independent effects of BKM-120, we examined the previously described potential effects of BKM-120 on microtubules^12^. In U251 cells, after 24 hours of BKM-120, the cells became rounded up with possible damage to the microtubule cytoskeleton (Fig. 3D). To further examine microtubule behavior we used U251 and U1242 cells expressing EB1-GFP. EB1 preferentially localizes to the plus-ends of growing microtubules and is pro-migratory in GBM^23^. A time-lapse study revealed a reduction in microtubule dynamics as indicated by EB1 trafficking after BKM-120 treatment, compared to a modest effect of GDC-0941 (Fig. 3D and Supplementary 3B). Due to the effects on cell adhesion observed above, we also investigated the effects of BKM-120 and GDC-0941 on focal adhesions using U251 cells expressing a paxillin-GFP fusion. This showed that 2 μM BKM-120 completely eliminated focal adhesions after 24 hours, whereas GDC-0941 had a much less pronounced effect (Fig. 3D). These data suggest that the effects of BKM-120 on the inhibition of GBM invasion and migration could be due to off-target effects on focal adhesions and microtubule dynamics.

### BKM-120 reduces GBM tumor spread *in vivo* in orthotopic xenografts

**Figure 4:**
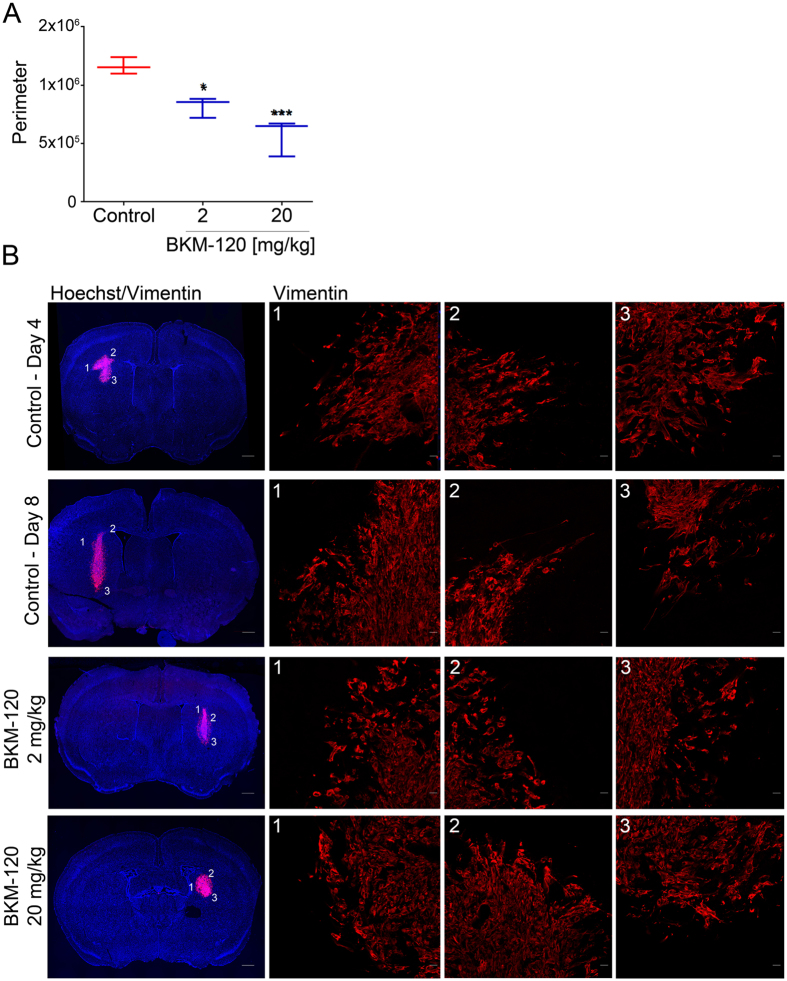
BKM-120 treatment blocks glioma growth and invasion *in vivo.* To analyze the effects of BKM-120 *in vivo*, we injected U1242 cells intracranially in nude mice and treated them daily with 2 mg/kg and 20 mg/kg of BKM-120 by gavage. (**A**) Average of the the tumors perimeters measured at day 7, and corrected for the averages of the size of the tumors at day 4. (**B**) Tumor spread was examined using Vimentin and DAPI staining. Photographs illustrate tumor growth (low resolution, bar = 1 mm) and the tumor normal brain interface indicating the degree of invasion (high resolution, bar = 20 μm). One-way ANOVA was used for statistical analysis (**p < 0.01, ***p < 0.001).

The effect of BKM-120 *in vivo* was examined using two orthotopic xenograft models. We performed a 10 day treatment in G9-copGFP cells and a 7 day study in U1242 cells. The G9-copGFP tumor-bearing mice (5 for each group) were treated with vehicle and BKM-120 20 mg/kg (Supplementary Fig. 3D). The U1242 tumor-bearing mice (5 for each group) were treated with vehicle and 2 different concentrations of BKM-120, 2 mg/kg and 20 mg/kg, corresponding to a human equivalent dose (HED) of 1.62 mg/kg and 0.16 mg/kg respectively (Fig. 4B). The tumor perimeter was measured after the treatments at day 7, and and the average tumor size was normalized to day 4 showing a strong and dose-dependent effect of BKM-120 *in vivo* (Fig. 4A). Microscopic analysis of tumor sections revealed a marked difference in tumor distribution compared with vehicle-treated control mice and high magnification analysis of tumors revealed a well-defined tumor/normal brain interface after treatment, and reduced migration (Fig. 4B and supplementary Fig. 3D).

Thus, BKM-120 has anti-invasive properties that alongside BBB penetration may render this a useful molecule for limiting GBM invasion. These effects are not mediated completely by PI3K inhibition, and future mechanistic studies will investigate the additional targets involved in these effects.

## Discussion

Our study shows that the CNS-penetrant PI3K inhibitor BKM-120 is a candidate anti-invasive drug for GBM treatment. BKM-120 inhibited invasion *in vitro* in a panel of GBM cell lines, in a range of assays, and this was not associated with cytotoxic effects. Moreover, BKM-120 is also able to inhibit invasion in neural stem cells and astrocytes at different concentrations, confirming the presence of common migratory mechanisms between cancer and normal cell lines. *In vivo* we were able to show reduced invasion in G9-copGFP and U1242 GBM cells after treatment with BKM-120.

PI3K isoforms have multiple roles in cell migration and, in particular, class IA and class IB subtypes are activated by, interact with, and contribute to activation of integrins and FAK^24^,^25^. However, all our GBM cell lines express PI3Kα and β isoforms and mostly all the others (Supplementary Fig. 4) and the effects of BKM-120 are likely to be directly on cell migration processes as opposed to invasion/remodelling, as the drug was equally as effective at blocking invasion in the presence and absence of matrix. The PI3K/Akt signaling pathway is activated by loss or mutation of the PTEN tumor suppressor. Previous papers support the PTEN-independence of the effects of BKM-120 on cell proliferation *in vitro*^10^,^11^. We also did not observe any correlation between BKM-120 and PTEN mutations (Supplementary Fig. 4). Most importantly in terms of the goal of this study, which was to investigate the potential of BKM-120 as an anti-invasive drug, orthotopic xenografts demonstrated a reduction in tumor spread and were less invasive after BKM-120 treatment. It should be noted that unequivocal monitoring of anti-invasive effects is challenging under conditions where growth is also slightly inhibited, as alterations in tumor metabolism in rapidly growing tumors may promote invasion^26^. In addition, BKM-120 treated tumors clearly show a more defined tumor/brain interface, which strongly supports our *in vitro* observations. Moreover, in contrast to high BKM-120 dose used for *in vivo* experiment by previous papers^10^,^12^,^27^, our experiments were performed using the mouse equivalent of the current maximum tolerated doses used in patients and a 10-times less concentrated dose.

Clinical trial data on recurrent GBM treated with BKM-120 are not yet published, but invasion was not part of these studies, so is unlikely to have been addressed in patients. Combination studies of BKM-120 may be critical to investigate its anti-invasive potential in human patients. It is of interest that a clinical trial is underway combining BKM-120 with avastin (NCT01349660). In the context of this combination, potential anti-invasive effects of BKM-120 may be addressed.

Interestingly, experiments with the similar PI3K inhibitor GDC-0941 showed that the effects of BKM-120 may be only partially mediated via PI3K inhibition. BKM-120, but not GDC-0941, strongly blocked invasion, and potently induced alterations in cell morphology, microtubule dynamics and eliminated focal adhesions. Further experiments are necessary to better understand direct and off-target effects of BKM-120, and to determine their relative contribution to the anti-migratory effects observed, and whether these are additive or synergistic with PI3K inhibition. Our data seems to indicate a direct effect on microtubule stability as previously observed^12^, given that the general structure of the microtubule network seems to be altered in our cells under conditions that block migration, however further experiments are necessary to elucidate the exact mechanism. EB1 tracking of microtubule plus ends revealed potential effects on microtubule dynamics. This may be related to the enhanced effect of BKM-120 on focal adhesions compared with GDC-0941. Others have established a link between microtubules and focal adhesion regulation, which may be relevant to our observations^24^,^25^,^28^. The differential effects of BKM-120 and GDC-0941 on GBM invasion suggest that class I PI3K functions in GBM migration, but also that class I PI3K inhibition alone is not sufficient to fully block cell motility. However, given that both BKM-120 and GDC-0941 are pan-class I inhibitors and we found a partial effect of PI3K on cell migration, further experiments will be necessary to underline the importance of each isoform and test them generating a drugs resistant allele^29^. There is data showing that Src^30^ and Akt kinases^6^ play a role in glioblastoma invasion. Although Src inhibition blocks migration in our cell line panel (data not shown), we did not see any changes in Src activation in the phospho-ELISA assay (Supplementary Fig. 3) which we have confirmed by Western blotting (data not shown). The observation that GDC-0941 does not block migration would rule out a role for Akt in our observations on GBM migration.

Overall, these support the important role of the off-target and direct effects of BKM-120 that are not yet fully defined. It is likely that further global profiling studies may uncover additional protein or lipid kinases that could underlie these observations. At present, the nature of the effects which may be involved in mediating some of the effects of BKM-120 is not clear, therefore elucidation of the underlying mechanism is of importance and may lead to the development of improved anti-invasive approaches.

In summary, the CNS-penetrant drug BKM-120 is able to inhibit GBM cell invasion *in vitro* and *in vivo*, and its potential anti-invasive properties should be investigated clinically in GBM and potentially other invasive tumor types. Further studies are necessary to completely define additional targets of BKM-120.

## Additional Information

**How to cite this article**: Speranza, M.-C. *et al.* BKM-120 (Buparlisib): A Phosphatidyl-Inositol-3 Kinase Inhibitor with Anti-Invasive Properties in Glioblastoma. *Sci. Rep.* **6**, 20189; doi: 10.1038/srep20189 (2016).

## Supplementary Material

Supplementary Information

## References

[b1] Cloughesy T. F., Cavenee W. K. & Mischel P. S. Glioblastoma: From Molecular Pathology to Targeted Treatment. Annu Rev Pathol. 9, 1–25 (2013). 23937436 10.1146/annurev-pathol-011110-130324

[b2] Ostrom Q. T. *et al.* CBTRUS Statistical Report: Primary Brain and Central Nervous System Tumors Diagnosed in the United States in 2007–2011. Neurooncol. 16, 1–63 (2014). 10.1093/neuonc/nou223 PMC4193675 25304271

[b3] Stupp R. *et al.* Radiotherapy plus concomitant and adjuvant temozolomide for glioblastoma. N. Engl. J. Med. 352, 987–96 (2005). 15758009 10.1056/NEJMoa043330

[b4] McCarty J. H. Glioblastoma resistance to anti-VEGF therapy: Has the challenge been MET? Clin. Cancer Res. 9, 1631–33 (2013). 23403631 10.1158/1078-0432.CCR-13-0051 PMC3618531

[b5] Dunn G. P. *et al.* Emerging insights into the molecular and cellular basis of glioblastoma. Genes Dev. 15, 756–84 (2012). 22508724 10.1101/gad.187922.112 PMC3337451

[b6] Wen P. Y., Lee E. Q., Reardon D. A., Ligon K. L. & Alfred Yung W. K. Current clinical development of PI3K pathway inhibitors in glioblastoma. Neuro Oncol. 14, 819–29 (2012). 22619466 10.1093/neuonc/nos117 PMC3379803

[b7] Rodon J. *et al.* Phase I dose-escalation and -expansion study of buparlisib (BKM120), an oral pan-Class I PI3K inhibitor, in patients with advanced solid tumors. Invest. New Drugs. 32, 670–81 (2014). 24652201 10.1007/s10637-014-0082-9

[b8] Brennan C. W. *et al.* The somatic genomic landscape of glioblastoma. Cell. 155, 462–77 (2013). 24120142 10.1016/j.cell.2013.09.034 PMC3910500

[b9] Bleeker F. E. *et al.* Mutational profiling of kinases in glioblastoma. BMC Cancer. 14, 718 (2014) 25256166 10.1186/1471-2407-14-718 PMC4192443

[b10] Wachsberger P. R. *et al.* Hsp90 inhibition enhances PI-3 kinase inhibition and radiosensitivity in glioblastoma. J. Cancer Res. Clin. Oncol. 140, 573–82 (2014). 24500492 10.1007/s00432-014-1594-6 PMC11823890

[b11] Koul D. *et al.* Antitumor activity of NVP-BKM120-A selective pan class I PI3 kinase inhibitor showed differential forms of cell death based on p53 status of glioma cells. Clin. Cancer Res. 18, 184–95 (2012) 22065080 10.1158/1078-0432.CCR-11-1558 PMC3785365

[b12] Brachmann S. M. *et al.* Characterization of the Mechanism of Action of the Pan Class I PI3K Inhibitor NVP-BKM120 across a Broad Range of Concentrations. Mol Cancer Ther. 11, 1747–57 (2012). 22653967 10.1158/1535-7163.MCT-11-1021

[b13] Cain R. J. & Ridley A. J. Phosphoinositide 3-kinases in cell migration. Biol. Cell. Biol. 101, 13–29 (2009). 10.1042/BC20080079 19055486

[b14] Mao P. *et al.* Mesenchymal glioma stem cells are maintained by activated glycolytic metabolism involving aldehyde dehydrogenase 1A3. Proc Natl Acad Sci USA 110, 8644–9 (2013). 23650391 10.1073/pnas.1221478110 PMC3666732

[b15] Williams S. P. *et al.* Indirubins decrease glioma invasion by blocking migratory phenotypes in both the tumor and stromal endothelial cell compartments. Cancer Res. 71, 5374–80 (2011). 21697283 10.1158/0008-5472.CAN-10-3026 PMC4288480

[b16] Nowicki M. O. *et al.* Lithium inhibits invasion of glioma cells; possible involvement of glycogen synthase kinase-3. Neuro. Oncol. 10, 690–9 (2008). 18715951 10.1215/15228517-2008-041 PMC2666245

[b17] Peruzzi P. *et al.* MicroRNA-128 coordinately targets Polycomb Repressor Complexes in glioma stem cells. Neuro Oncol. 15, 1212–24 (2013). 23733246 10.1093/neuonc/not055 PMC3748913

[b18] Agudelo-Garcia P. A. *et al.* Glioma Cell Migration on Three-dimensional Nanofiber Scaffolds Is Regulated by Substrate Topography and Abolished by Inhibition of STAT3 Signaling 1,2. Neoplasia. 13, 831–40 (2011). 21969816 10.1593/neo.11612 PMC3182275

[b19] Shannon R. S., Minakshi N. & Nihal A. Dose translation from animal to human studies revisited. FASEB J. 22, 659–661 (2008). 17942826 10.1096/fj.07-9574LSF

[b20] Center for Drug Evaluation and Research & Center for Biologics Evaluation and Research. Estimating the safe starting dose in clinical trials for therapeutics in adult healthy volunteers. U.S. Food and Drug Administration Rockville, Maryland, USA (2002).

[b21] Folkes A. J. *et al.* The identification of 2-(1H-indazol-4-yl)-6-(4-methanesulfonyl-piperazin-1-ylmethyl)-4-morpholin-4-yl-thieno[3,2-d]pyrimidine (GDC-0941) as a potent, selective, orally bioavailable inhibitor of class I PI3 kinase for the treatment of cancer. J. Med. Chem. 51, 5522–32 (2008). 18754654 10.1021/jm800295d

[b22] Sarker D. *et al.* First-in-human phase I study of pictilisib (GDC-0941), a potent pan-class I phosphatidylinositol-3-kinase (PI3K) inhibitor, in patients with advanced solid tumors. Clin Cancer Res. 21, 77–86 (2015). 25370471 10.1158/1078-0432.CCR-14-0947 PMC4287394

[b23] Berges R. *et al.* End-binding 1 protein overexpression correlates with glioblastoma progression and sensitizes to Vinca -alkaloids *in vitro* and *in vivo*. Oncotarget. 5, 12769–87 (2014). 25473893 10.18632/oncotarget.2646 PMC4350359

[b24] Ginsberg M. H., Partridge A. & Shattil S. J. Integrin regulation. Curr. Opin. Cell Biol. 17, 509–16 (2005). 16099636 10.1016/j.ceb.2005.08.010

[b25] Sulzmaier F. J., Jean C. & Schlaepfer D. D. FAK in cancer: mechanistic findings and clinical applications. Nat. Rev. Cancer. 14, 598–610 (2014). 25098269 10.1038/nrc3792 PMC4365862

[b26] Robertson-Tessi M., Gillies R. J., Gatenby R. A. & Anderson A. R. Impact of metabolic heterogeneity on tumor growth, invasion, and treatment outcomes. Cancer Res. 75, 1567–79 (2015). 25878146 10.1158/0008-5472.CAN-14-1428 PMC4421891

[b27] El Meskini R. *et al.* A preclinical orthotopic model for glioblastoma recapitulates key features of human tumors and demonstrates sensitivity to a combination of MEK and PI3K pathway inhibitors. Dis. Model. Mech. 3, 652–62 (2015). 10.1242/dmm.018168 PMC4283649 25431423

[b28] Yue J. *et al.* Microtubules regulate focal adhesion dynamics through MAP4K4. Dev. Cell. 31, 572–85 (2015). 25490267 10.1016/j.devcel.2014.10.025 PMC4261153

[b29] Zunder E. R., Knight Z. A., Houseman B. T., Apsel B. & Shokat K. M. Discovery of drug-resistant and drug-sensitizing mutations in the oncogenic PI3K isoform p110 alpha. Cancer Cell. 14, 80–92 (2008). 10.1016/j.ccr.2008.06.014 PMC2720137 18691552

[b30] Ahluwalia M., Groot J., Liu W. & Gladson C. L. Targeting SRC in glioblastoma tumors and brain metastases: rationale and preclinical studies. Cancer Lett. 298, 139–49 (2010). 20947248 10.1016/j.canlet.2010.08.014 PMC3212431

