# Supplementary material for: BKM-120 (Buparlisib): A Phosphatidyl-Inositol-3 Kinase Inhibitor with Anti-Invasive Properties in Glioblastoma
Source: Sci Rep. 2016 Feb 5;6:20189. doi: 10.1038/srep20189 (PMC4742861; doi:10.1038/srep20189)
Supplement: Supplementary Information [file srep20189-s1.pdf]

Supplementary information for

BKM-120 (Buparlisib): A Phosphatidyl-Inositol-3 Kinase Inhibitor with Anti-Invasive Properties in Glioblastoma by Maria-Carmela Speranza, Michal O. Nowicki, Prajna Behera, Choi-Fong Cho, E. Antonio Chiocca, Sean E. Lawler

SUPPLEMENTARY 1

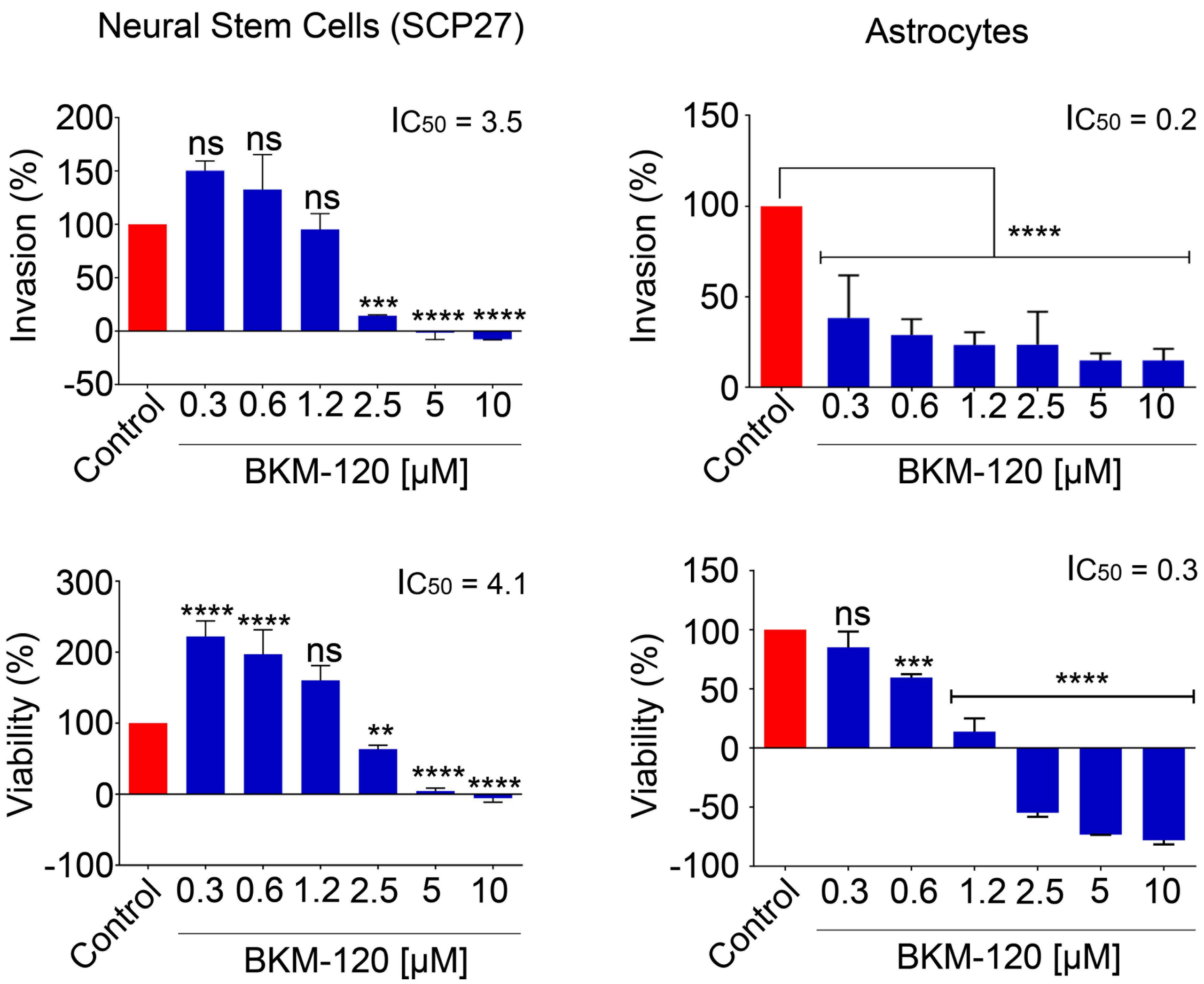

SUPPLEMENTARY 2

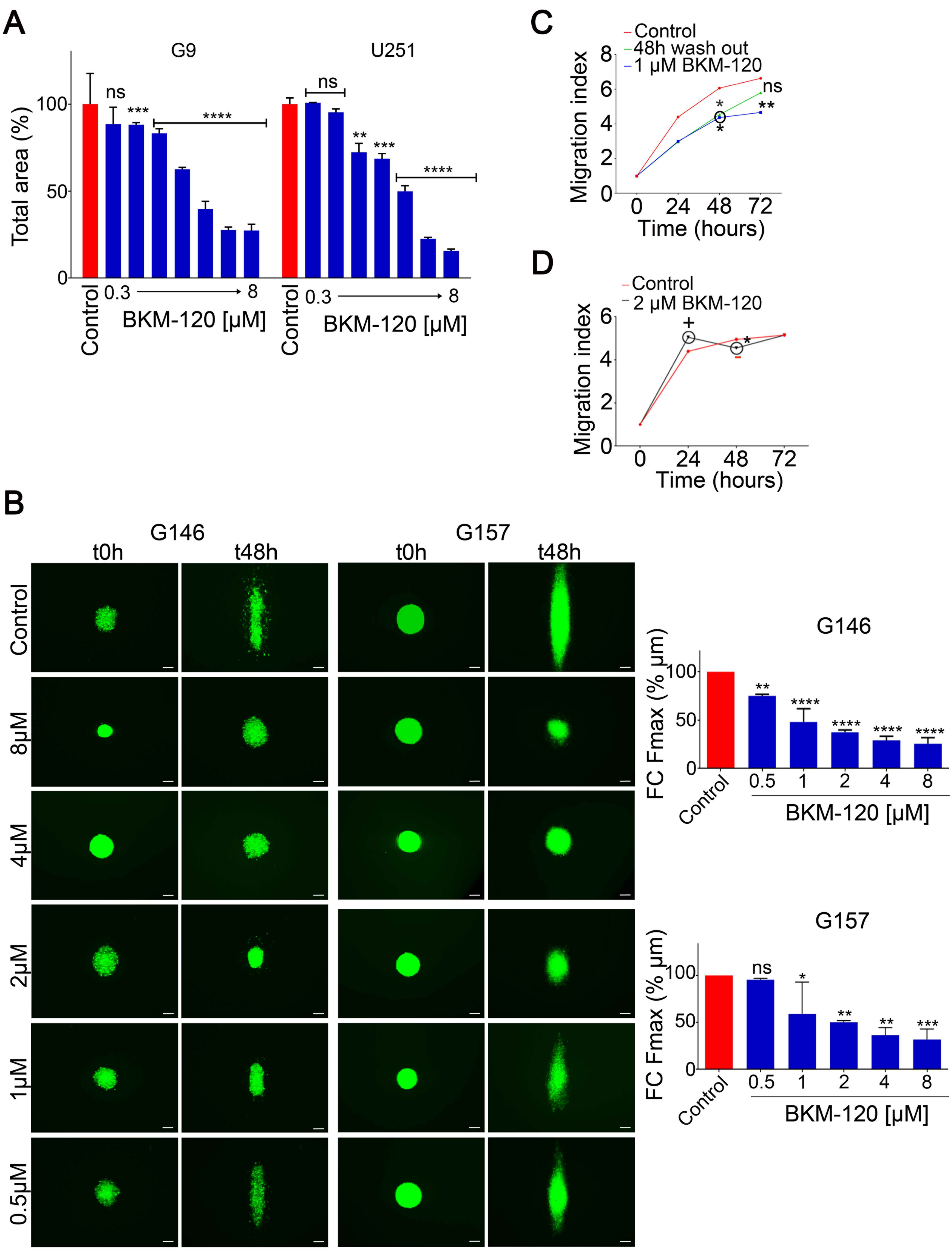

SUPPLEMENTARY 3

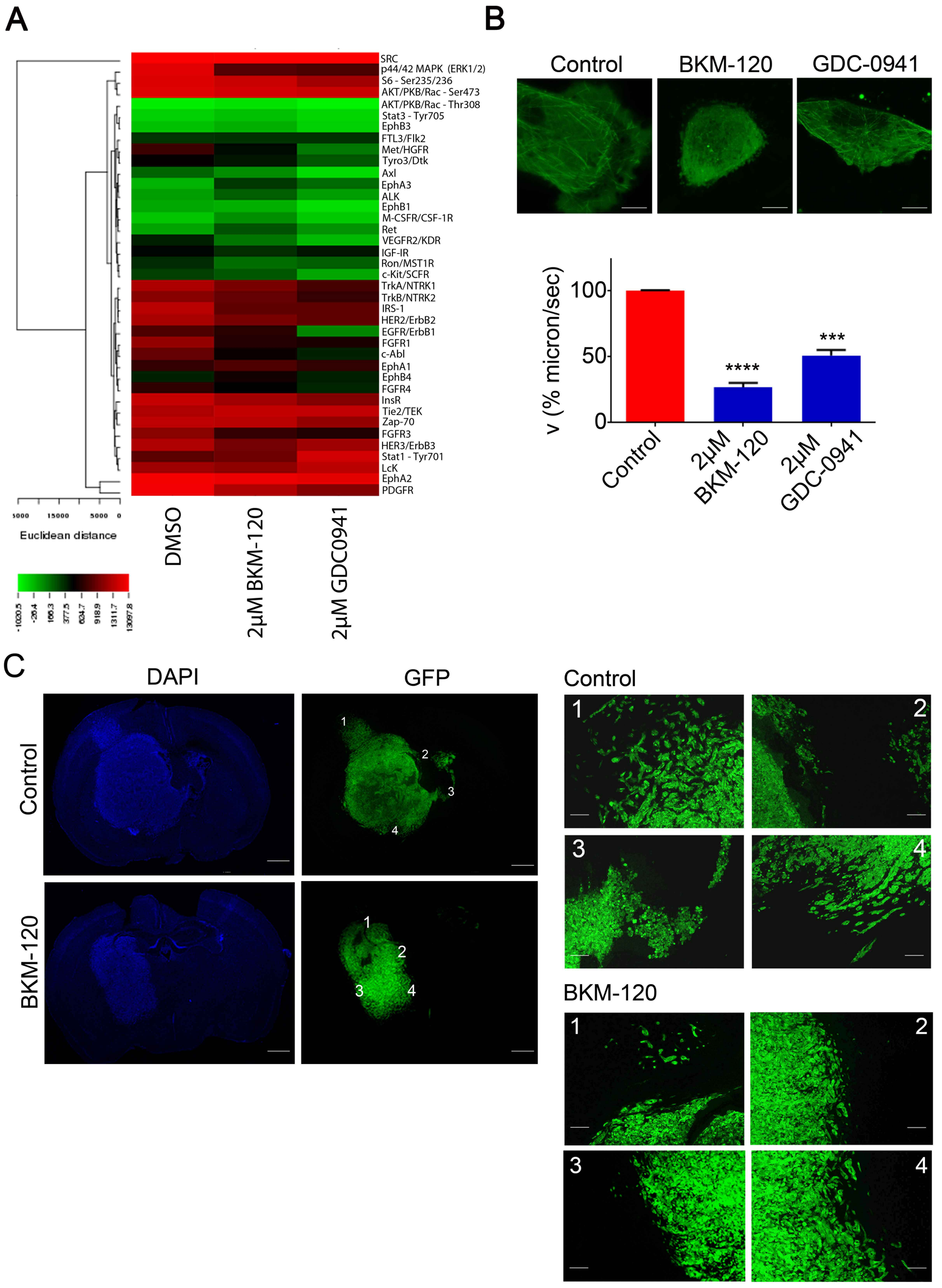

SUPPLEMENTARY 4

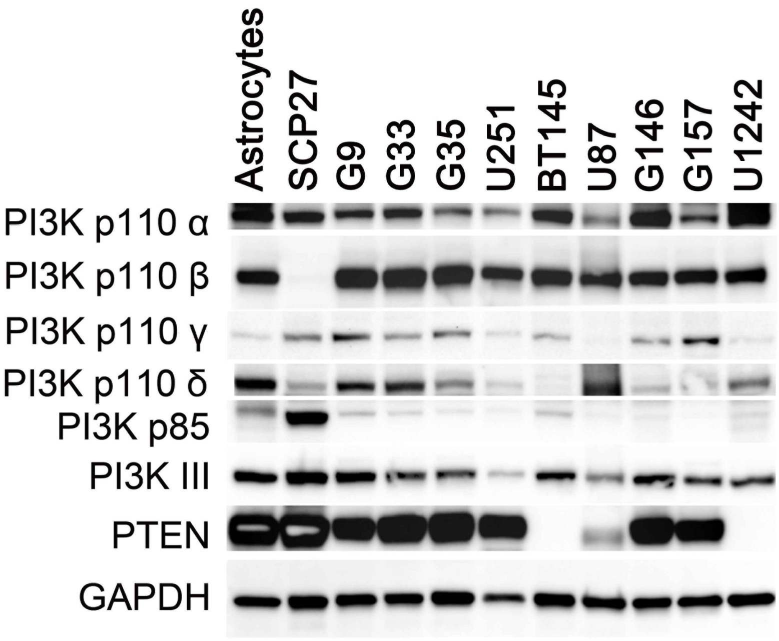

SUPPLEMENTARY TABLE 1

	1µM GDC-0941		1µM BKM-120	
Kinase	*% activity remaining*	*SD*	*% activity remaining*	*SD*
PI3K a E542K-p85	1	0	3	1
PI3Ka E545K-p85	1	0	3	0
PI3K b	4	0	12	0
PI3K a	4	0	6	0
PI3K g	36	6	51	7
SPHK1	55	23	75	15
CHK a	66	11	77	2
PIK4CB	80	12	83	1
SPHK2	82	26	78	17
CHK b	90	0	85	5
PI4K2a	95	11	97	1
DGK g	96	12	104	14
DGK z	97	4	102	5
DGK b	101	2	111	11
PIP5K2A	104	11	111	8

SUPPLEMENTARY TABLE 2

1µM GDC-0941			1µM BKM-120		
Kinase	*% activity remaining*	*SD*	*Kinase*	*% activity remaining*	*SD*
ABL	nd	nd	ABL	92	10
AMPK	76	8	AMPK	100	20
ASK1	83	2	ASK1	105	7
Aurora A	94	12	Aurora A	110	18
Aurora B	82	9	Aurora B	90	13
BRK	nd	nd	BRK	93	17
BRSK1	92	8	BRSK1	102	6
BRSK2	87	0	BRSK2	106	9
BTK	79	4	BTK	110	20
CAMK1	82	11	CAMK1	137	4
CAMKK beta	80	0	CAMKK beta	nd	nd
CAMKKb	nd	nd	CAMKKb	99	4
CDK2-Cyclin A	103	6	CDK2-Cyclin A	98	7
CDK9-Cyclin T1	nd	nd	CDK9-Cyclin T1	131	6
CHK1	85	3	CHK1	95	8
CHK2	99	5	CHK2	92	17
CK1γ2	nd	nd	CK1γ2	93	10
CK1δ	83	2	CK1δ	103	2
CK2	105	3	CK2	94	16
CLK2	30	2	CLK2	60	1
CSK	83	10	CSK	118	28
DAPK1	77	11	DAPK1	129	8
DDR2	nd	nd	DDR2	98	5
DYRK1A	92	0	DYRK1A	79	11
DYRK2	63	3	DYRK2	110	9
DYRK3	85	3	DYRK3	74	4
EF2K	98	4	EF2K	103	2
EIF2AK3	nd	nd	EIF2AK3	101	4
EPH-A2	100	11	EPH-A2	114	17
EPH-A4	107	8	EPH-A4	96	5
EPH-B1	101	9	EPH-B1	103	9
EPH-B2	94	1	EPH-B2	100	5
EPH-B3	92	0	EPH-B3	99	3

EPH-B4	88	12	EPH-B4	103	29
ERK1	93	10	ERK1	105	2
ERK2	72	3	ERK2	98	21
ERK5	nd	nd	ERK5	98	13
ERK8	79	1	ERK8	97	2
FGF-R1	91	12	FGF-R1	106	28
GCK	83	4	GCK	92	19
GSK3 beta	69	6	GSK3b	97	10
HER4	77	8	HER4	107	22
HIPK1	83	2	HIPK1	105	15
HIPK2	52	2	HIPK2	89	6
HIPK3	81	8	HIPK3	97	20
IGF-1R	84	0	IGF-1R	96	4
IKK beta	87	0	IKK beta	89	15
IKK epsilon	83	3	IKK epsilon	116	4
IR	93	7	IR	88	19
IRAK1	nd	nd	IRAK1	97	1
IRAK4	75	1	IRAK4	102	13
IRR	81	15	IRR	94	4
JAK2	83	4	JAK2	99	3
JNK1	97	3	JNK1	101	10
JNK2	83	3	JNK2	99	6
JNK3	72	4	JNK3	93	10
Lck	100	6	Lck	119	31
LKB1	52	5	LKB1	108	19
MAP4K3	nd	nd	MAP4K3	93	10
MAP4K5	nd	nd	MAP4K5	110	11
MAPKAP-K2	82	2	MAPKAP-K2	92	21
MAPKAP-K3	83	1	MAPKAP-K3	106	10
MARK1	97	1	MARK1	95	3
MARK2	79	6	MARK2	95	10
MARK3	99	0	MARK3	91	12
MARK4	93	1	MARK4	85	0
MEKK1	92	3	MEKK1	96	25
MELK	72	15	MELK	109	2
MINK1	76	1	MINK1	104	1
MKK1	80	12	MKK1	106	1

MKK2	82	0	MKK2	100	6
MKK6	77	10	MKK6	99	1
MLK1	74	3	MLK1	82	6
MLK3	74	8	MLK3	88	14
MNK1	95	4	MNK1	92	4
MNK2	91	2	MNK2	97	12
MPSK1	nd	nd	MPSK1	102	10
MSK1	72	1	MSK1	96	3
MST2	98	11	MST2	97	13
MST3	nd	nd	MST3	108	14
MST4	93	2	MST4	97	1
NEK2a	78	9	NEK2a	102	5
NEK6	100	7	NEK6	97	1
NUAK1	79	4	NUAK1	94	14
OSR1	nd	nd	OSR1	95	5
p38a MAPK	78	4	p38a MAPK	113	12
p38b MAPK	90	1	p38b MAPK	94	9
p38d MAPK	89	9	p38d MAPK	95	13
p38g MAPK	96	5	p38g MAPK	98	9
PAK2	95	5	PAK2	114	3
PAK4	85	0	PAK4	111	14
PAK5	80	0	PAK5	107	29
PAK6	89	2	PAK6	93	14
PDGFRA	nd	nd	PDGFRA	91	7
PDK1	90	9	PDK1	100	5
PHK	98	7	PHK	95	21
PIM1	82	13	PIM1	103	2
PIM2	87	10	PIM2	92	3
PIM3	87	0	PIM3	94	2
PINK	nd	nd	PINK	87	10
PKA	103	3	PKA	104	2
PKBa	89	7	PKBa	96	9
PKBb	63	8	PKBb	95	16
PKCa	99	10	PKCa	102	15
PKCz	82	3	PKCz	93	5
PKCγ	nd	nd	PKCγ	104	2
PKD1	79	10	PKD1	104	6

PLK1	96	5	PLK1	90	6
PRAK	100	6	PRAK	93	21
PRK2	71	6	PRK2	88	13
RIPK2	84	1	RIPK2	85	4
ROCK 2	73	2	ROCK 2	108	16
RSK1	86	2	RSK1	120	23
RSK2	82	0	RSK2	104	0
S6K1	81	2	S6K1	106	33
SGK1	93	6	SGK1	100	0
SIK2	nd	nd	SIK2	104	5
SIK3	nd	nd	SIK3	101	2
SmMLCK	70	9	SmMLCK	80	8
Src	76	6	Src	91	12
SRPK1	90	1	SRPK1	97	8
STK33	nd	nd	STK33	93	20
SYK	100	7	SYK	90	1
TAK1	80	8	TAK1	83	25
TAO1	83	5	TAO1	80	7
TBK1	80	0	TBK1	102	4
TESK1	nd	nd	TESK1	112	15
TGFBR1	nd	nd	TGFBR1	74	20
TIE2	nd	nd	TIE2	91	13
TLK1	nd	nd	TLK1	105	10
TrkA	67	6	TrkA	92	12
TSSK1	nd	nd	TSSK1	100	10
TTBK1	nd	nd	TTBK1	100	1
TTBK2	nd	nd	TTBK2	89	17
TTK	79	1	TTK	87	2
ULK1	nd	nd	ULK1	95	13
ULK2	nd	nd	ULK2	96	4
VEGFR1	74	12	VEG-FR	90	3
WNK1	nd	nd	WNK1	114	2
YES1	84	2	YES1	87	8
ZAP70	nd	nd	ZAP70	102	1

Supplementary Legends

Supplementary Table 1. Panel of 15 lipid kinases. The table indicates kinase name, the percent activity remaining and the standard deviation for each kinase after treatment with 1 μM of GDC-041 and 1 μM BKM-120. Experiments were performed at the International Centre for Kinase Profiling, Dundee, UK.

Supplementary Table 2. Panel of 140 protein kinases. The table indicate the kinase name, the % of activity remained and the standard deviation for each kinase after treatment with 1 μM of GDC-041 and 1 μM BKM-120. Experiments were performed at the International Centre for Kinase Profiling, Dundee, UK.

Supplementary Fig. 1. Effect of BKM-120 on neural stem cells and astrocytes. Dose-dependent effect of BKM-120 on invasion and cell viability/proliferation analysis of neural stem cells (SCP27) and astrocytes after 48 hours. IC50 was calculated using data from a range of BKM-120 concentrations.

Supplementary Fig. 2. BKM-120 inhibits G9-copGFP, U251-copGFP, G146-copGFP and G157-copGFP GSC migration in a dose-dependent manner and its effect is reversible. A, The graph shows a dose-dependent effect of BKM-120 on U251-copGFP and G9-copGFP in wound-healing assays at 48 h. B, Time course over 48 hours on nanofiber scaffolds of G146 and G157 GBM cells treated with 8, 4, 2, 1 and 0.5 μM BKM-120. The graphs indicate percentage of migration index compared to controls at 48 hours (100%) (bar = 100 μm). C, Time course of

migration over 72 hours in the presence of 1 μM BKM-120. The drug wash out performed at 48 hours demonstrates recovery of cell migration after drug removal. Control in red, 1 μM of BKM-120 in blue, BKM-120 wash out at 48h is shown in green. D, Time course over 72 hours of G9-copGFP GSCs. Control in red, BKM-120 in black. Spheres were treated with 2 μM BKM-120 (+) at 24h and wash out (-) was performed at 48h. All the experiments were performed in triplicate. ImageJ was used for the quantification. Control always treated with DMSO. One-way ANOVA was performed with Prism6 software (ns, not significant, *p<0.05, **p<0.01, ***p<0.001, ****p<0.0001).

Supplementary Fig. 3. Effect of BKM-120 and GDC-0941 on 39 phospho-proteins, microtubule dynamics and *in vivo* studies. A, ELISA-based screen of 39 major phospho-proteins (Cell Signaling, #7949). G157 GBM cells were treated for 30 minutes with BKM-120 or GDC-0941 (2 μM). Control was treated with DMSO. One matrix CIMminer (Weinstein, et al., Science 1997; 275:343-349) was used to build the heatmap. B, U1242-EB1-GFP time-lapse (bar = 10 μm) at 6h after DMSO, BKM-120 and GDC-0941 (2 μM). The velocity (microns/sec) of EB1 was quantified with ImageJ. C, G9-copGFP cells were intracranially injected in nude mice and treated daily with 20 mg/kg of BKM-120 by gavage. Tumor spread was examined using GFP and DAPI staining. Photographs illustrate tumor growth (low resolution, bar = 1 mm) and the tumor normal brain interface indicating the degree of invasion (high resolution, bar = 20 μm). The measurements were made in triplicate. One-way ANOVA was performed with Prism6 software (ns not significant, *p<0.05, **p<0.01, ****p<0.0001).

Supplementary Fig. 4. PI3K isoform and PTEN expression in GSCs. PI3K p110 α/β/δ/γ, PI3K p85, PI3K III (Cell Signaling #9655), PI3K p110 δ (Santa Cruz sc-7176) and PTEN (Cell Signaling #9552) expression levels were measured by Western blot. GAPDH was used as housekeeping gene. The measurements were made in triplicate. One-way ANOVA was performed with Prism6 software (ns not significant, *$p<0.05$, **$p<0.01$, ****$p<0.0001$).
